# Rheumatoid Arthritis: Its Pathology, Morbid Anatomy, and Treatment

**Published:** 1897-03

**Authors:** 


**Rheumatoid Arthritis: its Pathology, Morbid Anatomy, and Treatment.** By Gilbert A. Bannatyne, M.D. Pp. xii., 173. Bristol: John Wright & Co. 1896.

The discovery by Drs. Bannatyne and Wohlmann of a micro-organism of constant characteristics in the synovial fluid of cases of rheumatoid arthritis has thrown much light on the hitherto obscure etiology of this disease.

Dr. Bannatyne has rapidly followed this up by writing a fairly complete description on the subject from the new standpoint. The most important practical result brought forward in the book is, that treatment by internal antiseptics, such as guaiacol carbonate and benzosol, has led to most encouraging results. The chief point in which perhaps fault might be found is that there is evidence of some hastiness in bringing out such a treatise: for instance, the pathology of the muscular atrophy which so often occurs is discussed twice over, the second time, in the course of a long extract from an article contributed by the author to the *Lancet*.

